# High sensitivity troponin-I threshold to predict perioperative myocardial infarction

**DOI:** 10.1186/s13019-023-02323-0

**Published:** 2023-07-17

**Authors:** Tom Friedman, Dror B. Leviner, Veronica Chan, Bobby Yanagawa, Ady Orbach, Abd El Haleem Natour, Anastasia Weis, Erez Sharoni, Gil Bolotin

**Affiliations:** 1 Department of Cardiac Surgery, Rambam Health Care Campus, Haifa, Israel; 2 Department of Cardiothoracic Surgery, Carmel Medical Center, Haifa, Israel; 3 Division of Cardiac Surgery, St Michael’s Hospital, University of Toronto, Toronto, ON Canada; 4 Department of Cardiology, Rambam Health Care Campus, Haifa, Israel

## Abstract

**Background:**

High-sensitivity Troponin I (hs-cTnI) has largely replaced conventional troponin assays in an effort to improve detection of myocardial infarction. However, the mean displacement of hs-cTnI following coronary artery bypass graft (CABG) and the optimal threshold to detect perioperative myocardial infarction (MI) is unclear. Our objective is to describe mean hs-cTnI values at 6–12 h post-CABG and to determine the highest specificity while maintaining 100% sensitivity hs-cTnI cut-off values for diagnosis of perioperative or type-5 MI.

**Methods:**

Between 2016 and 2018, 374 patients underwent non-emergent, isolated CABG. Pre-operative and 6 h post-operative hs-cTnI values were recorded as well as ECG, echocardiographic and angiographic data.

**Results:**

Of 374 patients, 151 (40.3%) had normal and 224 (59.7%) had elevated preoperative hs-cTnI. Patients with normal preoperative hs-cTnI had a mean 6 h hs-cTnI of 9193 ng/l or 270X the upper normal value. Eleven patients (7.3%) presented with post-operative MI with a mean 6 h hs-cTnI of 50,218 ng/l or 1477X the upper normal value. Patients with elevated preoperative hs-cTnI had a mean 6 h hs-cTnI of 9449 ng/l or 292X the upper normal value. Eleven patients (4.9%) who presented with post-operative MI had a mean 6 h hs-cTnI of 26,823 ng/l or 789X the upper normal value.

**Conclusions:**

We suggest hs-cTnI threshold of 80-fold in patients with normal pre-operative hs-cTnI and 2.7-fold in patients with elevated pre-operative hs-cTnI. These results have important implications for perioperative care and for surgical trial reporting.

**Supplementary Information:**

The online version contains supplementary material available at 10.1186/s13019-023-02323-0.

## Introduction

Troponin assays have led to a revolution in the diagnosis of patients with myocardial infarction (MI). With the evolution of troponin assays to newer generations, we see a rise in sensitivity, with the most recent high-sensitivity troponins (hs-cTn) able to detect minimal myocardial injury. This higher sensitivity has led to rapid adoption of hs-cTn from its first introduction in Europe a little more than 10 years ago. In a survey from 2016, use of hs-cTn was around 60% in European hospitals and roughly 70% in the UK [1]. Its main utility is in the Emergency department where it has allowed for a more streamlined approach for either exclusion and discharge of patients after ruling out an acute coronary syndrome and earlier diagnosis of patients who do have an MI, leading to earlier triage, treatment, and admission [2].

It’s adoption in cardiac surgery, on the other hand, has not been as enthusiastic. A recent study examined the levels of hs-cTn in a wide verity of cardiac surgical procedures [3]. The authors concluded that “postoperative hs-cTnT release differed significantly depending on the type of cardiac surgery; (1) multiple variables can account for higher postoperative hs-cTnT release, complicating the clinical use of this parameter in the early postoperative setting; and (2) the literature-defined cut-off value for diagnosing CABG-related MI does not seem to be useful in a real-life clinical setting following different types of cardiac surgery.” leaving much to be desired.

Myocardial infarction post-coronary artery bypass grafting (CABG), also known as type 5 MI, can be the result of myocardial arrest, traumatic myocardial injury and due to acute ischemic injury secondary to technical failure. The diagnostic criteria for type 5 MI were defined by the 4th Universal Definition of Myocardial Infraction Task Force based on troponin elevation as well as electrocardiographic, angiographic or echocardiographic evidence [4, 5]. The troponin cut-off of 10 times the 99th percentile of the upper reference limit [URL] has been “arbitrarily selected” [4, 6, 7]. The 4th Universal Definition of Myocardial Infraction Task Force further recommends the use of high sensitivity cardiac troponin (hs-cTn) for the diagnosis of non-perioperative MI. They currently recommend using cardiac troponin (cTn) for type 5 MI primarily due to the lack of data to inform a cut-off threshold.

Documenting the rise in hs-cTnI post-CABG and the values in patients with confirmed type 5 MI is a critical unmet need. Here, we aim to describe the mean hs-cTnI values 6–12 h post-CABG in patients with normal and elevated preoperative hs-cTnI and to determine an optimal (i.e., with the highest specificity while maintaining 100% sensitivity) hs-cTnI cut-off value for the diagnosis of type 5 MI.

## Methods

### Patient selection

Between February 2016 and September 2018, patients who underwent isolated CABG at the Rambam Health Care Campus, Haifa, Israel were identified retrospectively. The trial protocol was approved by the hospital Research Ethics Board (0198-18). We included patients who had normal hs-cTnI before surgery and patients with elevated but stable or decreasing hs-cTnl before surgery. Exclusion criteria were emergent surgery, chronic dialysis and elevated and increasing preoperative hs-cTnI. Data included patient demographics, perioperative medications, and surgical data. Postoperatively, hs-cTnI was measured routinely 6 h after arrival in the intensive care unit, and then 12 and 24 h after surgery if clinically indicated. We analyzed 6 h hs-cTnI and the highest hs-cTnI level after surgery. A twelve-lead electrocardiogram (ECG) was performed routinely preoperatively and 1, 6, 24, and 48 h after surgery in the ICU.

### Hs-cTnl

The Abbott ARCHITECT STAT hs-cTnI immunoassay (Abbott inc., Abbott Park, IL) was utilized [8]. The 99th percentile URL of this assay is 15.6 pg/ml (ng/L) for females and 34 pg/ml (ng/L) for males. The 10% concentration of ARCHITECT STAT hs-TnI is 4.7 pg/mL (ng/L). For the 99th percentile URL for females, we used 16 pg/ml (ng/L), and 34 pg/ml (ng/L) for males.

### Perioperative MI definition

Perioperative MI was diagnosed based on 6 h hs-cTnI, ECG, echocardiography and/or angiography. After reviewing all patient data a consultant cardiologist decided whether the patient fulfilled the criteria of type 5 MI according to the 4th Universal Definition of Myocardial Infraction Task Force definition [4]:

- Elevation of hs-cTnI values > 10 times the 99th percentile URL in patients with normal baseline hs-cTnI values.
- OR Elevation of hs-cTnI values > 20% the baseline hs-cTnI in patients with an elevated baseline hs-cTnI (the absolute post-procedural hs-cTnI must be > 10 times the 99th percentile URL).

AND one of the following elements is required:

- New pathological Q waves
- Angiographically documented graft occlusion or new native coronary artery occlusion
- Imaging evidence of new loss of viable myocardium or new regional wall motion abnormality in a pattern consistent with an ischemic etiology

### Statistical analysis

All data were analyzed using SPSS software version 25. Descriptive statistics were produced using frequencies (N/%) for categorical variables (e.g., gender) and means with standard deviations for continuous variables. Differences between the study groups for the continuous variables were assessed using Mann–Whitney test, and for the discrete variables using the chi-square test.

## Results

### Patient characteristics

A total of 374 patients underwent isolated on-pump CABG (See Additional file 1: Table S1 for operative data). Of these, 151 (40.3%) had normal baseline hs-cTnl and 223 (59.7%) had elevated baseline hs-cTnl. Table 1 describes the baseline characteristics between the patients with and without type 5 MI. Patients with type 5 MI had a higher EuroSCORE II (5.3 ± 3.4 vs. 3.8 ± 4.0, *p* < 0.01). There were no significant differences in operative characteristics between groups.

**Table 1:** Patient characteristics

	No type-5 MI N = 352	Type-5 MI N = 22	All patients N = 374
	M ± SD	M ± SD	
Gender			
Female (N [%])	60 (17.0)	4 (18.1)	64 (17)
Male	292 (83.0)	18 (81.9)	310 (83)
Age (mean ± SD)	64.2 ± 10	65.81 ± 9.8	64.3 ± 10
Smoker			
Prior	53 (15.1)	7 (31.8)	60 (16)
Active	116 (33.0)	5 (22.7)	121 (32)
BMI	29.2 ± 4.9	29.1 ± 2.8	29.2 ± 4.8
Creatinine*	0.8 (0.72,1.1)	0.84 (0.8,1.4)	0.82 (0.79,1.2)
LVEF	52.2 ± 12.1	54.5 ± 12	52.3 ± 12.1
Hemoglobin	13.3 ± 1.9	13.4 ± 2	13.3 ± 1.9
EuroSCORE II*	2.4 (1.7, 4)	3.3 (1.9, 5)	2.4 (1.7, 4.1)
Hypertension	278 (79.0)	19 (86.4)	297 (79)
Hyperlipidemia	288 (81.8)	19 (86.4)	307 (82)
CRF	42 (12.0)	2 (9.1)	44 (11)
DM2	187 (53.1)	14 (63.6)	201 (54)
DM on insulin	115 (32.7)	11(50.0)	126 (34)
Chest pain	18 (5.1)	7 (31.8)	25 (7)
Acute coronary syndrome in the last 6 weeks	128 (36.4)	13 (68.4)	141 (38)
PVD	181 (51.4)	4 (19.0)	185 (49)
CVA/TIA	37 (10.5)	2 (9.1)	39 (10)
Atrial fibrillation	19 (5.4)	1 (4.5)	20 (5)
Acetylsalicylic acid	238 (67.6)	14 (63.6)	352 (94)
PY2I	92 (26.1)	6 (27.3)	98 (26)
Anticoagulation	28 (8.0)	1 (4.5)	29 (8)
Beta-blocker	173 (49.1)	10 (45.5)	183 (49)
Statin	237 (67.3)	14 (63.6)	251 (67)
ACE Inhibitors	161 (45.7)	6 (27.3)	167 (45)

Continuous data are shown as mean ± standard error, and categorical data as number (%)

*BMI* body mass index, *CRF* chronic renal failure, *CVA* cerebrovascular accident, *DM* diabetes mellitus, *EuroSCORE* European system for cardiac operative risk evaluation, *LVEF* left ventricular ejection fraction, *MI* myocardial infarction, *PVD* peripheral vascular disease, *TIA* transient ischemic attack

*Results are presented as Median and interquartile range (IQR)

Twenty-two patients (5.9%) were diagnosed with type 5 MI according to the 4th Universal Definition of Myocardial Infraction Task Force definition [4]. Of these, 15 (68.2%) had new Q-wave, six (27.3%) had new wall motion abnormalities on echocardiography and seven (31.8%) had graft occlusion verified angiographically (patients could have more than one abnormality, see Additional file 1: Table S2 for full post operative outcomes data).

### Hs-cTnI following CABG

Hs-cTnI was measured 6 h post-operatively in 100%, 12 h post-operatively in 40.9% and 24 h post-operatively in 20.1% (See Additional file 1: Table S3 for hs-cTnI data).

Patients with normal preoperative hs-cTnI had a mean 6 h hs-cTnI of 9193 ng/l or 270X the upper normal value. Notably, 98% of patients had post-operative hs-cTnI values > 10 times normal (4^th^ Universal Definition of Myocardial Infraction threshold). Eleven patients (7.2%) presented with post-operative MI with had a mean 6 h hs-cTnI of 50,218 ng/l or 1477X the upper normal value.

Patients with elevated preoperative hs-cTnI had a mean 6 h hs-cTnI of 9449 ng/l or 292X the upper normal value. Here, 75% of patients had both post-operative hs-cTnI values > 10 times normal and a hs-cTnI level more than 1.2 times the preoperative level (4th Universal Definition of Myocardial Infraction threshold). Eleven patients (4.9%) who presented with post-operative MI with had a mean 6 h hs-cTnI of 26,823 ng/l or 789X the upper normal value.

### Hs-cTnI threshold for perioperative MI

**Fig. 1:**
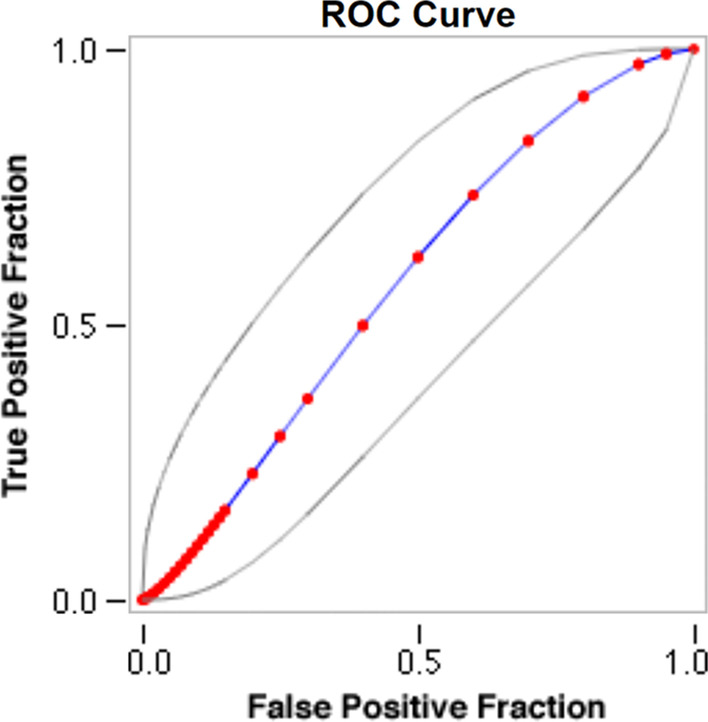
Type 5 MI ROC with peak hs-cTnI for patients with normal levels of hs-cTnI preoperatively, *hs-cTnI* high sensitivity cardiac troponin I, *ROC* receiver operating curve

**Fig. 2:**
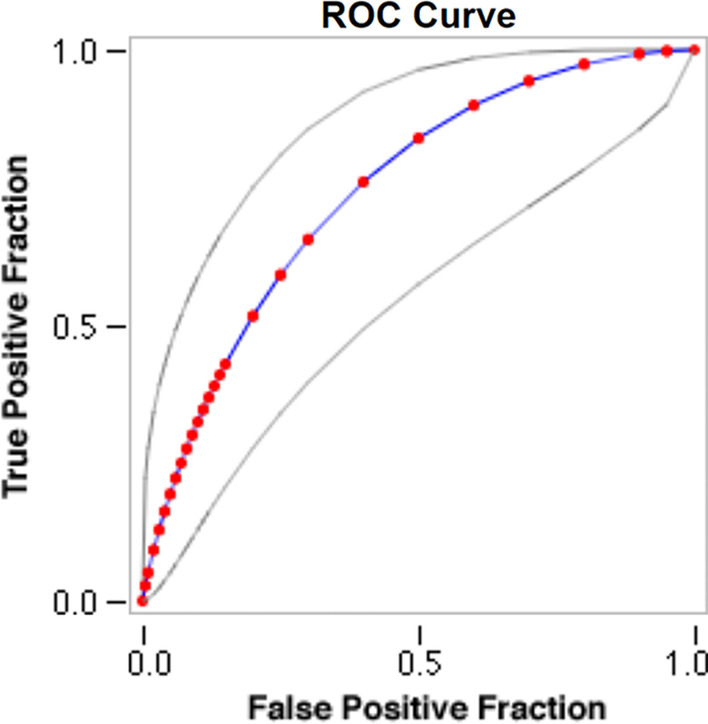
Type 5 MI ROC with peak hs-cTnI for patients with elevated stable levels of hs-cTnI preoperatively, *hs-cTnI* high sensitivity cardiac troponin I, *ROC* receiver operating curve

Sensitivity, specificity, and the receiver operating curve (ROC) were calculated for patients with and without a perioperative MI. For patients with normal preoperative hs-cTnl, a tenfold increase cut-off yielded sensitivity of 100% but specificity was 2.1% (ROC area under the curve (AUC) = 0.577, Fig. 1). By increasing the cut-off to an 80-fold increase, the specificity increased 6.5-fold from 2.1 to 13.2% (Fig. 2, Table 2).

**Table 2:** ROC curve and diagnostic performance of hs-cTnI for identification of Type 5MI for patients with normal and elevated baseline hs-cTnI

	Peak hs-cTnI at 24 h	Fitted ROC AUC	Optimal cut off	Sensitivity (%)	Specificity (%)	Positive predictive value (%)	Negative predictive value (%)
Normal Baseline hs-cTnI	Before optimization	0.577	tenfold	100	2.1	7.4	100
	After optimization		80-fold	100	13.2	8.4	100
Elevated Baseline hs-cTnI	Before optimization	0.745	1.2-fold	100	25.3	6.5	100
	After optimization		2.7-fold	100	40	12.3	100

*hs-cTnI* high sensitivity cardiac troponin I, *ROC* receiver operating curve

**Fig. 3:**
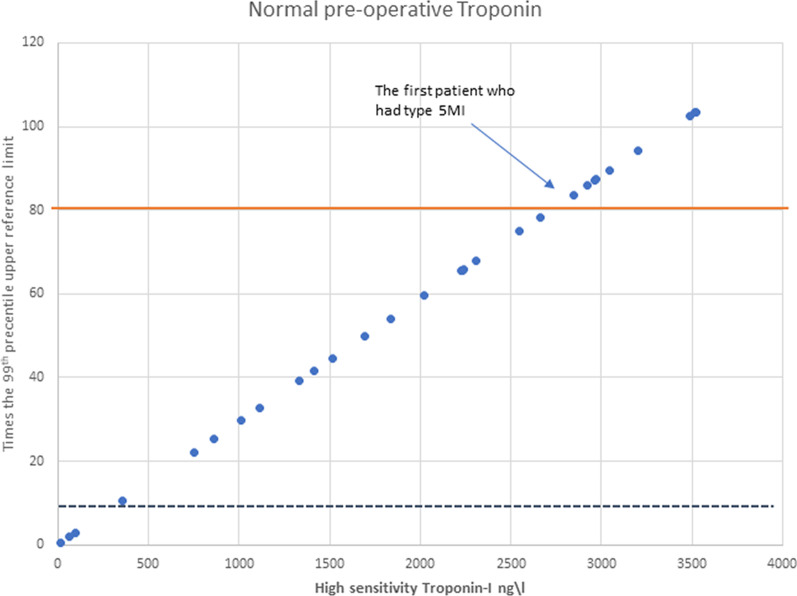
Scatter diagram of old versus new type 5MI threshold lines in patients with normal preoperative hs-cTnI. By increasing the cut off from 10 times the 99th percentile URL (blue dashed line) to 80 times the 99th percentile URL (orange continuous line) we increased specificity without losing sensitivity

**Fig. 4:**
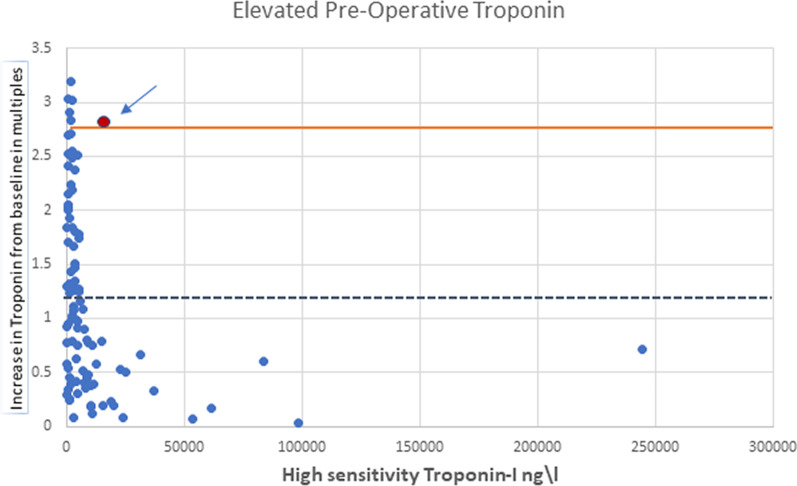
Scatter diagram of old versus new type 5MI threshold lines in patients with elevated preoperative hs-cTnI. In patients with an elevated level of hs-cTnl preoperatively, an increase to a cut off of 2.7-fold the base line hs-cTnl (orange continuous line) from 1.2-fold (blue dashed line) resulted in a twofold increase in specificity without losing sensitivity

For patients with elevated preoperative hs-cTnl, 1.2 times increase from preoperative levels as a cut-off yielded a sensitivity of 100% but specificity of 25.3% (ROC AUC = 0.745, Fig. 3). By increasing the cut-off from 1.2 to 2.7-fold, the specificity increased from 25.3 to 40.0% (Fig. 4, Table 2).

### Comment

**Fig. 5:**
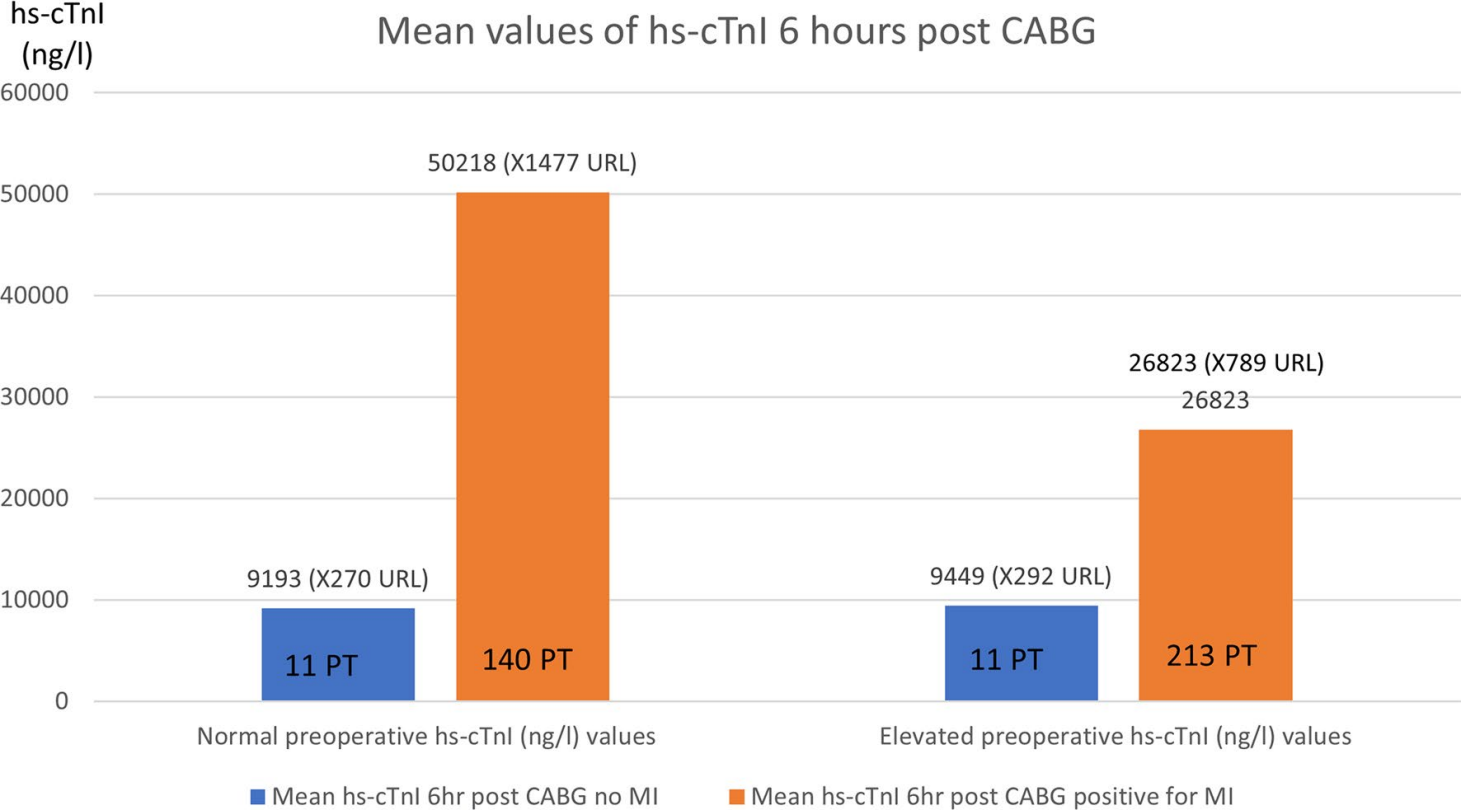
Number of patients with normal pre-CABG hs-cTnI and Elevated pre-CABG hs-cTnI groups with mean hs-cTnI results 6 h post CABG displayed for Type 5 MI (±)

In this study we report the following: (1) In patients with normal preoperative hs-cTnl, the mean values increased to 9193 ng/L and 50,2018 ng/L without and with perioperative MI, respectively (Fig. 5). (2) In patients with elevated preoperative hs-cTnl, the mean values increased to 9449 ng/L and 26,823 ng/L without and with perioperative MI, respectively. (3) The current 4th Universal Definition of Myocardial Infraction for type 5 MI offers poor specificity. (4) We suggest using 80-fold and 2.7-fold thresholds for type 5 MI in patients with normal and elevated hs-cTnl, respectively.

In 2012, the Third Universal Definition of Myocardial Infarction defined cardiac troponin instead of CK-MB as the preferable biomarker to diagnose myocardial necrosis [9]. The definition of MI associated with percutaneous coronary intervention (Type-4a MI), is as elevation of cTn > 5 times the 99th percentile URL is well established [4]. On the other hand, the cut-off of troponin used to define a myocardial infarction after CABG is “arbitrarily” and based on little data [4, 5]. Meanwhile, most cardiac centers have shifted to the use of hs-cTn [10]. The advantages being enhanced precision at low concentrations [11], which in turn begets a quicker diagnostic evaluation [12].

There remains controversy regarding the optimal biomarker cut-off for type 5 MI. In earlier studies, cut-off values range from > 35 to > 165 times the 99th percentile URL [6, 13–15]—much higher than the > 10 times the 99th percentile URL used in the current definition. In a recent publication [16], demonstrated that among patients who underwent isolated CABG or aortic-valve replacement or repair, the threshold troponin level, measured on post operative day 1 that was associated with an adjusted hazard ratio of more than 1.00 for death within 30 days was 5670 ng per liter (95% confidence interval [CI], 1045–8260), a level 218 times the upper reference limit. Our results are similar to this large database.

The current definition also distinguishes between patients with normal troponin preoperatively and elevated troponin preoperatively. However, this distinction is not well defined since the cut-off is identical between the two groups and they only differ by the added value or 1.2X times the preoperative level in the elevated group. In this study we distinguished between the two situations: patients with normal and elevated preoperative hs-cTnl. Since the clinical benefits of using hs-cTnI have been well established [17–19] in other clinical scenarios, we aimed to describe its utility in the setting of type 5 MI.

Based on these results and previous studies [5, 14, 15, 20] it appears that the current guidelines proposed by the fourth universal definition are not setting a reasonable threshold for separation between patients who have had an MI post-surgery and those who have not. The incidence of type 5 MI varies depending on the diagnostic procedure used. When assessed by elevation in cardiac biomarkers and evidence of ECG changes, the incidence has been reported to range from 5 to 14% [21]. When using cardiac magnetic resonance (CMR) to detect new loss of viable myocardium, the incidence ranges from 20 to 30% [14, 22].

There are important clinical and trial-related implications for determining a biomarker threshold for perioperative MI. The 5-year results of the EXCEL trial showed that the risk of death, stroke, or MI (the study’s primary endpoints) was 22.0% in the PCI arm and 19.2% in CABG-treated patients, a difference that was not statistically significant (*p* = 0.13). As a result, the EXCEL investigators concluded there was no significant difference between revascularization with PCI or CABG in patients with left main coronary artery disease of low or intermediate anatomical complexity [23]. It was later noted that the only endpoint in favor of PCI at 30 days was a reduction in myocardial infarction (3.9% with PCI vs. 6.3% with surgery; HR 0.63; 95% CI 0.42–0.94), which was driven by periprocedural events. At the 30 days–1 year time point there was no significant advantage from either procedure, but outcomes in the 1–5-year time frame favored CABG surgery. In a letter to the editor [24] Taggart et al. claim that the investigators used a new, untested definition of periprocedural myocardial infarction that clearly penalizes surgery, namely type 5MI. This definition is the key driver of the composite outcome that does not lead to a significant difference in the two treatment strategies. In our opinion the type 5MI definition is not valid and robust enough to be used as a valid predictor for perioperative MI as it has been previously manipulated in the EXCEL trial.

### Limitations

This was a retrospective study with some inherent limitations. The definition of patients as having type 5 MI was based on clinical judgment and so we might have missed patients who had a type 5 MI without significant clinical sequela. This was also a single center study on a relatively small number of patients with only on pump CABG and a small number of events. The study described the differences in Troponin levels of two main groups—positive and negative for type 5 MI but did not describe the clinical results of those patients.

## Conclusion

This study is one of few studies, to the best of our knowledge, which examine hs-cTnI values after CABG, to try and validate new cut-off values for hs-cTnI for the diagnosis of type 5 MI. Setting the cut off value in patients with normal hs-cTnl preoperatively at 80 times the RUL results in a substantial increase in specificity without losing sensitivity. In patients with an elevated level of hs-cTnl preoperatively, an increase of 270% in hs-cTnl resulted in a twofold increase in specificity, again, without losing sensitivity. However, even after increasing the threshold substantially, sensitivity and specificity remain rather low. We thus believe that our data, combined with the other studies mentioned earlier, put the role of hs-cTnI in AMI after CABG into question and other biomarkers must be sought.

## Supplementary Information

**Additional file 1**. **Table S1**: Operative characteristics. **Table S2**: Post-operative characteristics. **Table S3**: hs-cTnI levels, pre- and post-surgery divided by two characteristics hs-cTnI levels before surgery: normal levels versus elevated levels.

## Data Availability

Data and materials can be obtained by contacting the author by email: t_friedman@rambam.health.gov.il

## Abbreviations

AUC: Area under the curve
cTn: Cardiac troponin
CABG: Coronary artery bypass grafting
CK-MB: Creatine kinase-MB
ECG: Electrocardiogram
Hs-cTn: High sensitivity cardiac troponin
ICU: Intensive care unit
MI: Myocardial infarction
ROC: Receiver operating curve
URL: Upper reference limit
