# Supplementary material for: High sensitivity troponin-I threshold to predict perioperative myocardial infarction
Source: J Cardiothorac Surg. 2023 Jul 17;18:234. doi: 10.1186/s13019-023-02323-0 (PMC10351123; doi:10.1186/s13019-023-02323-0)
Supplement: Supplementary file 1 — Additional file 1. Table S1: Operative characteristics. Table S2: Post-operative characteristics. Table S3: hs-cTnI levels, pre- and post-surgery divided by two characteristics hs-cTnI levels before surgery: normal levels versus elevated levels. [file 13019_2023_2323_MOESM1_ESM.docx]

**Table S1: Operative characteristics**

|  | **No type 5 MI** | | **Type 5 MI** | | **P** |
| --- | --- | --- | --- | --- | --- |
|  | **M±SD** | **N**  **(%)** | **M±SD** | **N**  **(%)** |  |
| **Number of grafts** | 3.05±1.12 |  | 3.32±1.13 |  | .13 |
| **Aortic cross-clamp (min)** | 54.91±32.41 |  | 60.23±30.45 |  | .18 |
| **CBP (min)** | 82.96±46.73 |  | 86.41±42.01 |  | .37 |
| **Graft to LAD** |  |  |  |  | .82 |
| **LIMA or RIMA** |  | 338  (96) |  | 22  (100) |  |
| **RADIAL** |  | 2  (0.6) |  | 0 |  |
| **SVG** |  | 12  (3.4) |  | 0 |  |
| **Second arterial graft** |  |  |  |  | .13 |
| - **LIMA or RIMA** |  | 128  (40.7) |  | 8  (40.0) |  |
| - **RADIAL** |  | 23  (7.3) |  | 0 |  |
| - **IABP** |  | 7  (2.0) |  | 1  (0.05) | .70 |

CBP - Cardiopulmonary bypass, IABP - Intra-aortic balloon pump, LAD - Left anterior descending, LIMA - Left internal mammary, RIMA - Right internal mammary, SVG - Saphenous vein graft

**Table S2: Post-operative characteristics**

|  | **No type 5 MI** | **Type 5 MI** | **P** |
| --- | --- | --- | --- |
|  | **M±SD/N (%)** | **M±SD/N**  **(%)** |  |
| **Creatinine** | 1.02±0.64 | 1.04±0.70 | .22 |
| **Acute renal failure** | 14 (4.0) | 0 | .34 |
| **CVA/TIA** | 29 (8.2) | 2 (9.1) | .89 |
| **Deep sternal wound infection** | 9 (2.6) | 1(4.5) | .58 |
| **Reoperation for bleeding** | 9 (2.6) | 0 | .45 |
| **Mortality within 30 days** | 8 (2.3) | 0 | .47 |
| **Hs-cTnl - number of patients at 6h** | 352 (98.6) | 22 (86.4) | < .01 |
| **Hs-cTnl – number of patients at 12h** | 144 (40.9) | 9 (40.9) | .99 |
| **Hs-cTnl – number of patients at 24h** | 67 (19.0) | 8 (36.4) | .049 |
| **Aspirin** | 297 (84.4) | 21 (95.5) | .16 |
| **DAPT** | 105 (29.8) | 7 (31.8) | .84 |
| **Anticoagulation** | 36 (10.2) | 0 | .11 |
| **Beta-blockers** | 304 (100) | 22 (100) | - |
| **Statin** | 261 (74.1) | 10 (45.5) | < .01 |
| **ACEI** | 154 (43.8) | 4 (18.2) | .02 |

CVA – Cerebrovascular accident, ACEI – Angiotensin converting enzyme inhibitors, DAPT – Dual antiplatlet therapy, hs-cTnI - High sensitivity cardiac troponin I, MI – Myocardial infarction, TIA – Transient ischemic attack

**Table S3: hs-cTnI levels, pre- and post-surgery divided by two characteristics hs-cTnI levels before surgery: normal levels vs elevated levels**

|  | **No type 5 MI**  **M±SD** | **N (%)** | **Type 5 MI**  **M±SD** | **N (%)** |
| --- | --- | --- | --- | --- |
| **Normal pre-surgery hs-cTnI** | 12.9±8.25 | 140 (93%) | 16.3±9.9 | 11 (7%) |
| **Max hs-cTnI post-surgery** | 5969±13499 |  | 50218±127582 |  |
|  |  |  |  |  |
| **Elevated pre-surgery hs-cTnI** | 5297±20050 | 212 (95%) | 706±841 | 11 (5.1%) |
| **Max hs-cTnI post-surgery** | 9073±20888 |  | 26823±43999 |  |

hs-cTnI - High sensitivity cardiac troponin I, MI – Myocardial infarction
